# Mcl-1 Ubiquitination and Destruction

**DOI:** 10.18632/oncotarget.242

**Published:** 2011-03-19

**Authors:** Hiroyuki Inuzuka, Hidefumi Fukushima, Shavali Shaik, Pengda Liu, Alan W. Lau, Wenyi Wei

**Affiliations:** Department of Pathology, Beth Israel Deaconess Medical Center, Harvard Medical School, Boston, MA 02215

**Keywords:** Ubiquitination, SCF, Fbw7, GSK3, Mcl-1, Apoptosis, Tumor Suppressor, Phosphorylation, Cell Cycle

## Abstract

Loss of the Fbw7 tumor suppressor is common in diverse human cancer types, including T-Cell Acute Lymphoblastic Leukemia (T-ALL), although the mechanistic basis of its anti-oncogenic activity remains largely unclear. We recently reported that SCF^Fbw7^ regulates cellular apoptosis by controlling the ubiquitination and destruction of the pro-survival protein, Mcl-1, in a GSK3 phosphorylation-dependent manner. We found that human T-ALL cell lines displayed a close relationship between Fbw7 loss and Mcl-1 overexpression. More interestingly, T-ALL cell lines that are deficient in Fbw7 are particularly sensitive to sorafenib, a multi-kinase inhibitor that has been demonstrated to reduce Mcl-1 expression through an unknown mechanism. On the other hand, Fbw7-deficient T-ALL cell lines are much more resistant to the Bcl-2 antagonist, ABT-737. Furthermore, reconstitution of Fbw7 or depletion of Mcl-1 in Fbw7-deficient cells restores ABT-737 sensitivity, suggesting that elevated Mcl-1 expression is important for Fbw7-deficient cells to evade apoptosis. Therefore, our work provides a novel molecular mechanism for the tumor suppression function of Fbw7. Furthermore, it provides the rationale for targeted usage of Mcl-1 antagonists to treat Fbw7-deficient T-ALL patients.

## INTRODUCTION

Dysregulated cell cycle progression leads to uneven distribution of the genetic information between the two daughter cells, which contributes to genomic instability and ultimately, cancer development. Recent work established that two related, multi-component E3 ubiquitin ligase enzymes, the Anaphase Promoting Complex (APC) and the Skp1-Cullin1-F-box complex (SCF), are the major driving forces governing proper cell cycle progression [1-4]. APC is active from the late G2 phase to mid-G1 phase, and is responsible for degradation of mitotic cyclins, securin and geminin [5, 6]. On the other hand, SCF is thought to be active from the late G1 phase until the G2 phase and mediates the ubiquitination and destruction of G1 cyclins and Cdk inhibitors [1, 7]. SCF consists of the adaptor protein Skp1, the scaffold protein Cul1, the ring-finger protein Rbx1, as well as a variable component that is responsible for substrate recognition known as the F-box protein. The human genome encodes 68 putative F-box proteins, thereby providing sufficient flexibility for substrate specificity [8]. Most of the physiological functions of these putative F-box proteins remain unknown. The well-characterized F-box proteins include Skp2, Cdc4/Fbw7, and β-TRCP1, which targets p27 [9], cyclin E [10], and Cdc25A [11], respectively, for ubiquitination and degradation. In all cases, proper phosphorylation of the substrate is required for its interaction with the F-box proteins.

## FBW7 IS A TUMOR SUPPRESSOR

**Figure 1:**
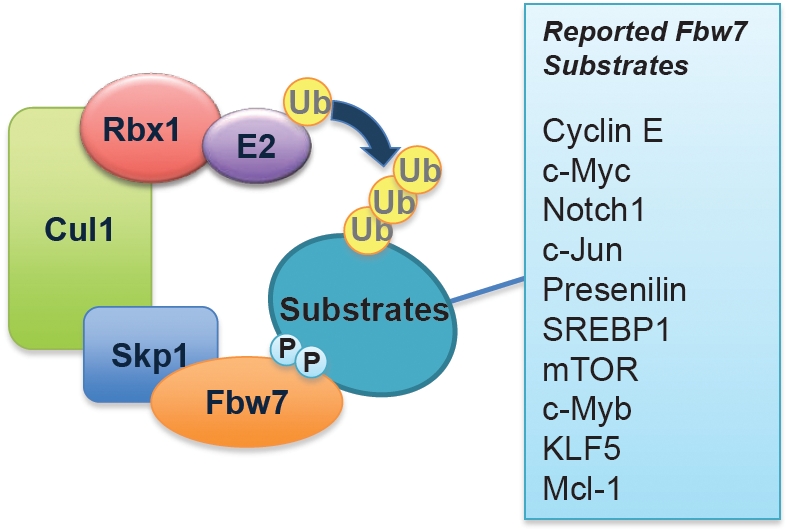
Schematic illustration of the SCF^Fbw7^ E3 ubiquitin ligase complex and a list of its identified downstream ubiquitin substrates

**Figure 2:**
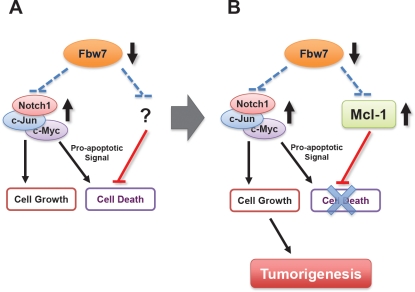
Fbw7 participates in the regulation of cellular apoptosis by targeting the pro-survival factor Mcl-1 for ubiquitination and destruction **A.** Loss of Fbw7 leads to the elevated expression of c-Myc, c-Jun, and the Notch-1 protein, all of which possess oncogenic functions and are frequently found to be overexpressed in various human cancers, including leukemia. Besides promoting cell growth, it has been shown that overexpression of either c-Jun, c-Myc or Notch-1 provokes cellular apoptosis. However, it remains unclear how Fbw7-deficient cells evade programmed cell death in the setting of upregulated c-Jun, c-Myc or Notch-1. **B.** We recently reported that loss of Fbw7 also leads to a significant elevation in Mcl-1 expression, which suppresses the induction of apoptosis by inactivating the pro-apoptotic function of many BH3 only proteins including Bim, Bax and Bak

Loss of Fbw7 is frequently observed in various types of tumors including breast cancer, colon cancer [12] and T-cell acute lymphoblastic leukemia (T-ALL) [13]. It has been documented that tissue-specific deletion of Fbw7 in mouse T cells results in the development of T-ALL [14-16], suggesting that Fbw7 is a novel tumor suppressor in T-ALL. However, the exact molecular mechanisms by which Fbw7 exerts its anti-tumor activity are still unknown [4]. We previously discovered that Fbw7 regulates the degradation of c-Jun in a GSK3 phosphorylation-dependent manner [17]. Our work assigned a biological significance to the v-Jun S243F point mutation and also underscored the importance of Fbw7 in tumor suppression [17]. In addition to the turnover of cyclin E [10] and c-Jun, Fbw7 is also involved in the degradation of c-Myc [18, 19], and the Notch-1 protein [20] (Figure 1), all of which have been reported to possess oncogenic functions and are frequently found to be overexpressed in various human cancers, including leukemia. Consistent with frequent loss of Fbw7, overexpression of c-Myc, c-Jun and Notch-1 is closely associated with the development of T-ALL. Besides accelerating cell growth [21], overexpression of either c-Jun, c-Myc or Notch-1 results in cell death through upregulation of the pro-apoptotic protein Bim-1 [22]. However, despite the ever-growing list of Fbw7 ubiquitin substrates (Figure 1), it remains unclear how Fbw7-deficient cells evade cell death in the setting of upregulated c-Jun, c-Myc or Notch-1 (Figure 2A).

## THE MCL-1 ONCOPROTEIN IS FOUND TO BE FREQUENTLY OVEREXPRESSED IN LEUKEMIA

Expression of the anti-apoptotic protein Mcl-1 is frequently elevated in various human tumors including leukemia, but the underlying mechanisms causing its elevation are not fully understood [23, 24]. Mcl-1 is a pro-survival member of the Bcl-2 family of proteins, which can suppress apoptosis by interacting with and suppressing the activities of pro-apoptotic proteins including Bim, Bax and Bak [25]. However, unlike other Bcl-2 family members, the Mcl-1 protein is extremely unstable, having a very short half-life [26]. The rapid induction and destruction of Mcl-1 has been proposed as a molecular mechanism for cells to switch into either the survival or apoptotic pathways in response to various stresses [27, 28]. Although GSK3 phosphorylation is reported to regulate Mcl-1 stability directly [26], little is known about the upstream E3 ubiquitin ligase that targets phosphorylated Mcl-1 for destruction. As illustrated in Figure 2B, we and others recently reported that Fbw7 targets Mcl-1 for ubiquitination and destruction in a GSK3-dependent manner [29, 30]. Therefore, our studies suggest that the simultaneous elevation of the pro-survival factor Mcl-1 provides a protection mechanism allowing Fbw7-deficient cells to evade apoptosis, thus providing a novel molecular mechanism for the tumor suppression function of Fbw7 (Figure 2B). Moreover, Mcl-1 has been demonstrated to play a key role in regulating the cellular apoptosis of T cells, but not other tissue types such as liver cells [27, 31]. Therefore, our studies also provide the possible mechanism for why loss of Fbw7 is very frequently observed in T-ALL patients.

## THE FBW7-DEFICIENT T-ALL CELLS ARE “ADDICTED” TO HIGH EXPRESSION LEVELS OF MCL-1

**Figure 3:**
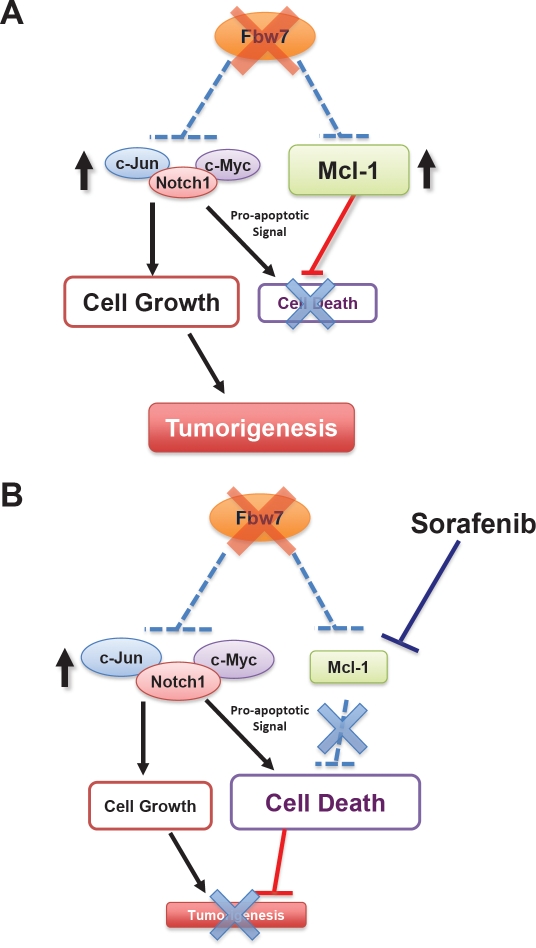
Fbw7-deficient T-ALL cells are “addicted” to high levels of Mcl-1 expression and are particularly sensitive to the Mcl-1 antagonist sorafenib **A.** In unstressed Fbw7-deficient T-ALL cells, induced expression of the pro-survival factor Mcl-1 balances the pro-apoptotic effects of elevated c-Jun, c-Myc and Notch-1 oncoproteins. However, the cells become “addicted” to high expression levels of the Mcl-1 oncoprotein. **B.** When Fbw7-deficient cells are treated with the Mcl-1 antagonist, sorafenib, the pro-survival function of Mcl-1 is inhibited. On the other hand, loss of Fbw7 leads to elevated expression of c-Jun, c-Myc and Notch-1, which provokes cellular apoptosis. When the anti-apoptotic effect of Mcl-1 is inhibited, cells undergo programmed cell death. Therefore, compared to the WT-Fbw7 T-ALL cells, Fbw7-deficient T-ALL cells are very sensitive to Mcl-1 antagonists.

Our studies for the first time provide firm experimental evidence for a potential role for the Fbw7 tumor suppressor in the modulation of the apoptotic pathway by governing Mcl-1 ubiquitination and destruction. Therefore, loss of Fbw7 not only provides a growth advantage by upregulating the c-Jun and c-Myc oncoproteins, but also leads to elevated Mcl-1 expression as a protection mechanism allowing Fbw7-deficient cells to evade possible apoptosis induced by high c-Jun and c-Myc expression (Figure 2B). However, this intricate balance to evade apoptosis seems to depend on high levels of the Mcl-1 oncoprotein. As a result, compared to WT-Fbw7 cells, Fbw7-deficient T-ALL cells are much more sensitive to the Mcl-1 inhibitor, sorafenib (Figure 3). Sorafenib is a multi-kinase inhibitor reported to suppress B-Raf, PDGF receptor and VEGF receptor kinase activities. Although its ability to repress Mcl-1 has been attributed to inactivating MAPK kinase and/or activating GSK-3 [32], the exact mechanism remains unclear. Nevertheless, this data suggests that Fbw7-deficient T-ALL cell lines might require high levels of the Mcl-1 oncoprotein to evade apoptosis, a phenotype that has been described previously as “oncogene addiction” [33]. Our studies thus provide a basis for personalized medicine for T-ALL patients as well as the rationale for developing specific Mcl-1 antagonists, or agents that significantly reduce Mcl-1 expression, to treat T-ALL patients.

## THE FBW7-DEFICIENT T-ALL CELLS ARE RESISTANT TO ABT-737 TREATMENT

**Figure 4:**
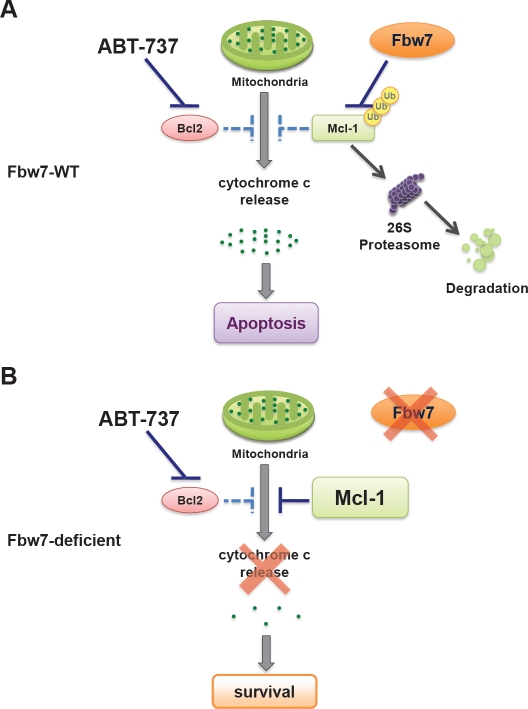
Loss of Fbw7 leads to elevated Mcl-1 expression, which confers increased resistance to the pan-Bcl-2 inhibitor ABT-737 **A.** In T-ALL cells with WT-Fbw7 genetic background, Mcl-1 stability is governed by Fbw7 in a GSK3-dependent manner. Inhibition of the Bcl-2 family of proteins with ABT-737 efficiently triggers apoptosis by inducing cytochrome c release. **B.** Due to structural differences, the pan-Bcl-2 inhibitor ABT-737 cannot efficiently inactivate Mcl-1 as it does to the rest of the Bcl-2 family of proteins. Therefore, loss of Fbw7 leads to elevated Mcl-1 expression, subsequently resulting in increased resistance to ABT-737.

The BH3 mimetic ABT-737 is a specific pan-inhibitor of the Bcl-2 family of anti-apoptotic proteins, which is reported to effectively kill leukemia cells, presumably by disrupting the Bcl2/Bax complex and inducing the Bak-dependent apoptotic pathway [34]. However, leukemia cells with elevated Mcl-1 expression are found to be refractory to ABT-737 treatment [35, 36] primarily because ABT-737 fails to inactivate Mcl-1 due to a low binding affinity [34]. Consistent with this, we found that Fbw7-deficient T-ALL cells, which displayed a significant increase in Mcl-1 expression, are much more resistant than Fbw7-WT T-ALL cells in response to ABT-737 (Figure 4). We further showed that depletion of Mcl-1, or reintroduction of Fbw7 into Fbw7-deficient T-ALL cells, restored ABT-737 sensitivity. This suggests that increased Mcl-1 expression is the determining factor that confers Fbw7-deficient cells resistance to ABT-737. Although it warrants further investigation, this work indicates that Fbw7-deficient T-ALL patients may not respond well to ABT-737 treatment.

## FBW7 IS THE PHYSIOLOGICAL E3 LIGASE THAT TARGETS MCL-1 FOR UBIQUITINATION IN T-ALL

Besides Fbw7, other E3 ubiquitin ligases including c-Mule [37] and β-TRCP [38] have also been implicated in Mcl-1 stability control. However, we found that although depletion of c-Mule leads to Mcl-1 upregulation in T-ALL, this regulation is not contingent upon GSK3-dependent phosphorylation of Mcl-1 [37, 38]. Most importantly, unlike the frequent loss of Fbw7 found in T-ALL, no correlation was found between the expression of c-Mule and Mcl-1 in various T-ALL cell lines. These results exclude a physiological role for c-Mule in regulating Mcl-1 in T-ALL cells. Additionally, although ectopic expression of β-TRCP promotes Mcl-1 destruction, no β-TRCP-dependent induction of Mcl-1 ubiquitination was observed. Furthermore, depletion of endogenous Fbw7, but not endogenous β-TRCP, leads to a significant induction of Mcl-1, rejecting the notion that β-TRCP physiologically control Mcl-1 abundance in T-ALL cell lines. Consistent with this finding, array CGH analysis demonstrated a high frequency of Fbw7 loss [13], but not simultaneous loss of β-TRCP1 and β-TRCP2 in the T-ALL cell lines. These data together support the hypothesis that the Fbw7 tumor suppressor, which is frequently lost in T-ALL, is the physiological E3 ubiquitin ligase for Mcl-1. However, it remains unclear whether in other tissue types or cellular context, other than Fbw7, c-Mule or β-TRCP will be the major force that governs Mcl-1 stability.

## WHETHER THE PTEN/PI3K/AKT AXIS IS THE UPSTREAM SIGNALING PATHWAY THAT GOVERNS FBW7-MEDIATED UBIQUITINATION OF MCL-1

**Figure 5:**
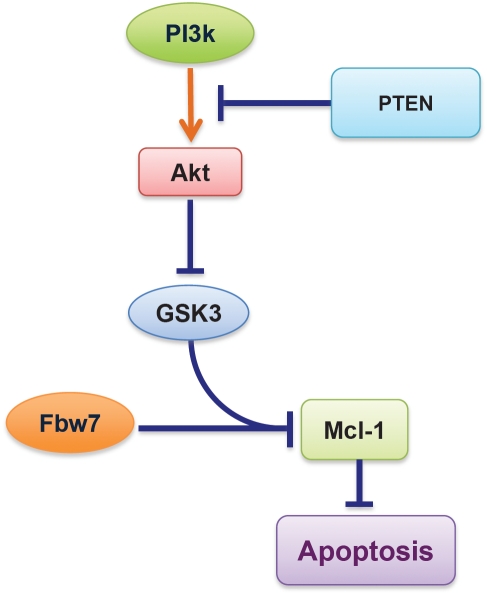
Schematic illustration of the potential role for the PTEN/PI3K/Akt signaling axis in governing Fbw7-mediated Mcl-1 ubiquitination and destruction Loss of PTEN, which leads to aberrant activation of the PI3K/Akt signaling cascade, results in inactivation of GSK3 and a subsequent increase in Mcl-1 expression.

Our work indicates that loss of Fbw7 contributes to Mcl-1 overexpression in T-ALL. However, only 20-30% of T-ALL cases are estimated to possess inactive Fbw7 [13], thus it is critical to understand how Fbw7-mediated Mcl-1 ubiquitination is physiologically regulated *in vivo*, and how Mcl-1 expression is aberrantly elevated in WT-Fbw7 genetic background, which accounts for 70-80% of T-ALL cases. To this end, we found that in WT-Fbw7 genetic backgrounds, loss of the PTEN tumor suppressor, which leads to inactivation of GSK3 kinase, also results in elevated Mcl-1 expression in a similar fashion to Fbw7-deficiency (data not shown). The PTEN phosphatase is a negative regulator of the PI3K/Akt signaling pathway and loss of PTEN is frequently observed in many types of tumors including T-ALL [13, 39-41]. Since our recent studies clearly demonstrated that GSK3 plays a critical role for Fbw7-mediated Mcl-1 ubiquitination, we hypothesize that any aberrant inactivation of GSK3, including loss of PTEN activity [42], might phenocopy Fbw7 deficiency, resulting in elevated Mcl-1 expression (Figure 5). Looking forward, it is thus critical to determine, especially in the setting of T-ALL, the molecular mechanisms by which the PTEN/PI3K/Akt/GSK3 axis participates in Fbw7-mediated destruction of Mcl-1, and to evaluate whether Akt or mTOR inhibitors could be a novel anti-leukemia therapeutic option to trigger apoptosis by decreasing Mcl-1 protein abundance, especially for those who are PTEN-deficient.
